# Specific PFKFB3 Inhibitor Memorably Ameliorates Intervertebral Disc Degeneration via Inhibiting NF-*κ*B and MAPK Signaling Pathway and Reprogramming of Energy Metabolism of Nucleus Pulposus Cells

**DOI:** 10.1155/2022/7548145

**Published:** 2022-09-21

**Authors:** Xiankun Cao, Xin Wang, Kewei Rong, Kexin Liu, Xiao Yang, Tangjun Zhou, Pu Zhang, Jiadong Guo, Hui Ma, An Qin, Jie Zhao

**Affiliations:** Shanghai Key Laboratory of Orthopedic Implants, Department of Orthopaedics Surgery, Shanghai Ninth People's Hospital, Shanghai Jiao Tong University School of Medicine, Shanghai 200011, China

## Abstract

Intervertebral disc (IVD) degeneration (IVDD) is a characteristic of the dominating pathological processes of nucleus pulposus (NP) cell senescence, abnormal synthesis and irregular distribution of extracellular matrix (ECM), and tumor necrosis factor-*α* (TNF-*α*) induced inflammation. Nowadays, IVD acid environment variation which accelerates the pathological processes mentioned above arouses researchers' attention. KAN0438757 (KAN) is an effective inhibitor of selective metabolic kinase phosphofructokinase-2/fructose-2,6-bisphosphatase 3 (PFKFB3) that has both energy metabolism reprogramming and anti-inflammatory effects. Therefore, a potential therapeutic benefit of KAN lies in its ability to inhibit the development of IVDD. This study examined *in vitro* KAN toxicity in NP primary cells (NPPs). Moreover, KAN influenced tumor necrosis factor-*α* (TNF-*α*) induced ECM anabolism and catabolism; the inflammatory signaling pathway activation and the energy metabolism phenotype were also examined in NPPs. Furthermore, KAN's therapeutic effect was investigated *in vivo* using the rat tail disc puncture model. Phenotypically speaking, the KAN treatment partially rescued the ECM degradation and glycolysis energy metabolism phenotypes of NPPs induced by TNF-*α*. In terms of mechanism, KAN inhibited the activation of MAPK and NF-*κ*B inflammatory signaling pathways induced by TNF-*α* and reprogramed the energy metabolism. For the therapeutic aspect, the rat tail disc puncture model demonstrated that KAN has a significant ameliorated effect on the progression of IVDD. To sum up, our research successfully authenticated the potential therapeutic effect of KAN on IVDD and declaimed its mechanisms of both novel energy metabolism reprogramming and conventional anti-inflammation effect.

## 1. Introduction

IVD usually plays an important role in spinal movement. As a result of IVDD, low back pain and severe spinal disorders, including lumbar disc herniation and spinal stenosis, are common [1]. Due to the wide range of morbidity, it has caused a huge socioeconomic burden and sorely influenced patients' quality of life [2]. The NP cell senescence and their dysfunction on ECM maintenance are considered as an initial trigger in the pathogenesis of IVDD [3]. The special physiological composition of hyaline cartilaginous endplates (CEPs), annulus fibrosus (AF), and NP determines the specific hypoxic environment of IVD. Therefore, AF rupture caused hypoxic environment disruption, and subsequent blood vessels ingrowth and nociceptive nerves [4] are the vital reasons for accelerated irregular distribution and abnormal synthesis of ECM and TNF-*α*-induced inflammation [5] and the continuous vicious circle of NP cell senescence. Under the circumstances, therapies targeting inflammation, senescence, and hypoxic microenvironment loss of NP cells [6–8], no matter by any kind of approaches such as EVs, cell secretory factors, stem cells, and drugs [9–11], have been the main assault fortified directions of researchers in last decades.

However, some emerging but interesting perspectives have aroused researchers' attention in the past few years. One of these hot issues, acid environment variation of degenerated IVD, similar to hypoxia environment change, has been demonstrated to influence some processes of IVDD [12, 13]. Specifically, the glycolytic metabolism phenotype of NP cells under a hypoxia environment is further aggravated due to the reduction of glucose and oxygen supply caused by CEP permeability decrease during IVDD, which enhances the lactate production and its efflux to ECM [14, 15]. The descending pH in the environment in turn significantly induces the catabolic upregulation and anabolic downregulation of acid-sensitive NP cells, resulting in the NP cells' senescence, ECM imbalance, and degradation [16]. The long-termed and persistently aggravated environment acidification not only further influences the CEP permeability and oxygen balance in hypoxia IVD but also accelerates the death of NP cells [17], which all contribute to the TNF-*α* production and expression of other inflammatory mediators [18]. Thus, therapies that effectively break this vicious circle and achieve the treatment of IVDD by regulating the IVD acidic environment are new directions worthy of further study.

PFKFB3 is a key glycolysis rate-limiting enzyme. Due to its strong kinase activity, stabilized expression of PFKFB3 can elevate the rate of glycolysis, thereby promoting the blood vessel sprouting, affecting tumor angiogenesis [19]. Therefore, targeting PFKFB3 of its effects on glycolysis of endothelial cells (ECs) during tumor angiogenesis has become a burgeoning research field in tumor therapy [20, 21]. In addition, the effect of PFKFB3 on endothelial cell glycolysis has also been demonstrated to be very important for pathological hypoxic diseases such as pulmonary hypertension [22]. Moreover, PFKFB3 inhibitor PFK15 exhibits a significant antiosteoporosis effect by repressing inflammation (NF-*κ*B and MAPK pathways) and blocking glycolysis of osteoclasts at the same time [23].

KAN is the latest inhibitor of selective metabolic kinase PFKFB3, more effective than PFK15, that has been identified as a potential cancer treatment strategy [24]. Therefore, according to previous research on the effect of PFKFB3 inhibition in ECs and OCs, whether and how KAN ameliorates IVDD, a hypoxia environment-metabolism dysfunction-NP cell senescence and death-inflammation vicious cycle pathological process, via inhibiting inflammation and reprogramming of energy metabolism of nucleus pulposus cells is worth to be explored and revealed.

## 2. Materials and Methods

### 2.1. Chemicals and Reagents

The selective metabolic kinase PFKFB3 inhibitor KAN0438757 (KAN) was purchased from MCE (CAS: 1451255-59-6, Shanghai, China), dissolved in dimethyl sulfoxide (DMSO) as a 5 mM stock solution, and stored at −80°C. A final concentration of DMSO less than 0.1% was used to reduce cytotoxicity. Fetal bovine serum (FBS) and penicillin/streptomycin were bought from Gibco BRL (Gaithersburg, MD, USA). Minimal essential medium (DMEM) was bought from Hyclone (Logan, UT, USA). The Cell Counting Kit-8 (CCK-8) was purchased from Dojindo Molecular Technology (Japan). The prime script RT Master Mix kit and TBGreen ® Premix Ex Taq ™ kit were both obtained from Takara Biotechnology (Otsu, Shiga, Japan). The XF Cell Mito Stress Test (for OCR) Kit, XF Glycolysis Stress Test (for ECAR) Kit, XFe96 FluxPak mini, XF DMEM Base Medium, XF 1.0M Glucose Solution, 100 mM XF Pyruvate Solution, and 200 mM XF Glutamine Solution were obtained from Agilent (Seahorse Bioscience, USA). The primary antibodies against MMP9, MMP13, and PFKFB3 were purchased from Abcam (Cambridge, UK). Primary antibodies including ERK, JNK, p38, phospho-ERK (Tr202/Tyr204), phospho-JNK (Tr183/Tyr185), phospho-p38, IKK*β*, phospho-IKK*α*/*β*, I*κ*B*α*, phospho-I*κ*B*α*, P65, phospho-P65, and *β*-actin and the secondary antibody were purchased from Cell Signaling Technology (CST, Danvers, MA, USA). All the antibodies mentioned except *β*-actin are rabbit anti-mouse.

### 2.2. NP Primary Cell (NPP) Isolation

Pentobarbital sodium 50 mg/kg (body weight) was injected intraperitoneally into 4-week-old SD rats for euthanasia. After that, the NP tissues were obtained from the caudal discs (Co1–Co6) and then digested for 2 hours by 0.25% type II collagenase at 37°C, 21% O_2_, and 5% CO_2_ incubator. Then, the mixture was centrifuged, and cells were separated, resuspended, and seeded (DMEM with 10% FBS and 1% penicillin-streptomycin) in a culture plate placing on 37°C, 21% oxygen, and 5% carbon dioxide incubator.

### 2.3. Cell Counting Kit-8 (CCK-8) Assay

The cell proliferation and toxicity effects of KAN on NPPs were measured using the CCK-8. More specifically, to determine toxicity, the NPPs were seeded triplicately into 96-well plates at 8 × 10^3^ cells per well in presence of various concentrations of KAN (control (0), 156.25, 312.5, 625, 1250, 2500, and 5000 nM) and cultured for 24 h. As for proliferation, 96-well plates were seeded with NPPs in triplicate at 2 × 10^3^ cells per well for 24 hours first. Then, KAN concentrations of control, 156.25, 312.5, 625, 1250, 2500, and 5000 nM were added into the wells for 24, 48, 72, and 96 hours. Cells were incubated at 37°C for 2 hours with 10 *μ*L of the CCK-8 reagent after each experimental period. The optical density values were measured on the Infinite M200 Pro multimode microplate reader using a spectrophotometer at 450 nm (Tecan Life Sciences, Männedorf, Switzerland).

### 2.4. High-Density Culture

To assess the ECM secretion ability effect of KAN, 10 *μ*l medium micromasses containing approximately 1.2 × 10^5^ NPPs were seeded in the middle at the bottom of a 24-well plate first. Then, the plate was placed in a 37°C, 21% O_2_, and 5% CO_2_ incubator for 90 min for the attachment of NPPs to the bottom. After that, 500 *μ*L of MEM/F12 medium (2% FBS and 10 ng/mL of Insulin Transferrin Selenium (ITS)) was added into the plate. The medium was changed every two days; These micromasses were stained with Alcian blue at the day 7.

### 2.5. Seahorse Metabolic Flux Analysis

NPPs were plated in XF-96 cell culture plates at 6 × 10^3^/well (Seahorse Bioscience, USA) for the first 24 hours. Then, the cells were treated with control, TNF-*α*, or TNF-*α* plus 2.5 *μ*M KAN for the next 24 hours. The measurements of oxygen consumption rate (OCR) and extracellular acidification rate (ECAR) were carried out according to standard protocols, and the results were detected with the Seahorse XF-96 Flux Analyzer (Seahorse Bioscience), as previously described [25]. In the OCR assay, 1.5 *μ*M oligomycin, 2.5 *μ*M FCCP, rotenone, and 0.5 *μ*M antimycin A were used to stimulate cells. In the EACR assay, 10 mM glucose, 2 *μ*M oligomycin, and 50 mM 2-DG were used to stimulate cells. Additionally, the OCR and ECAR changes were used to calculate OXPHOS characteristics as well as glycolysis parameters (as described in Supplementary Figure 1).

### 2.6. Immunofluorescence Assay

In a 6-well plate, 2 × 10^5^ per well NPPs was seeded and cultured for 12 hours. After that, the treatment group was pretreated with the serum-free DMEM in the presence of 2.5 *μ*M KAN for 2 hours; then, 15 min 20 ng/mL TNF-*α* stimulation, 10 min paraformaldehyde (4%) fix, and 10 min 0.2% Triton X-PBS permeabilization were performed on NPPs. The phospho-p65 primary antibody (1 : 200 dilution, Cell Signaling Technology, Danvers, MA, USA) was then incubated with cells overnight at 4°C after 10% goat serum blocking. After three times washing with PBS on the next day, the cells were incubated at dark with the Alexa Fluor 594 conjugate anti-rabbit secondary antibody for 1 hour (1 : 500, Cell Signaling Technology, Danvers, MA, United States). The cell nucleus was shown by 4′,6-diamidino-2-phenylindole (DAPI) after 3 min incubation (Sigma-Aldrich, St. Louis, MO, United States). Finally, digital fluorescence results were observed, and images were captured by the Leica fluorescence microscope (Olympus, Inc., Tokyo, Japan).

### 2.7. Protein Extraction and Western Blot (WB) Analyses

To figure out the effect of KAN on the catabolic or anabolic proteins, 3 × 10^5^ cells/well NPPs were cultured in DMEM on 6-well plates for 24 hours. The NPPs were then treated with control, TNF-*α*, or TNF-*α* plus 2.5 *μ*M KAN for 1 day. As for the effect of KAN on the phosphorylated proteins (short-time activated), 5 × 10^5^ cells/well NPPs were cultured in DMEM on 6-well plates for 24 hours. The treatment group cells were then pretreated with 2.5 *μ*M KAN for 2 hours with serum-free DMEM. 2 hours later, 10 and 30 min of TNF-*α* (20 ng/mL) stimulation were performed on the TNF-*α* group and the treatment group. After that, RIPA lysis buffer plus phosphatase and protease inhibitors (1 : 100 dilution, Roche, Basel, Switzerland) were used to, respectively, extract total cellular proteins at indicated time points. According to the standard protocol of the bicinchoninic acid protein quantification kit, protein concentrations were then quantified (Thermo Fisher Scientific, Waltham, MA, United States). Standard western blot experiments were performed as previously described [26]. Primary antibodies (*β*-actin, 1 : 1000; ERK, 1 : 1000; JNK, 1 : 1000; p38, 1 : 1000; IKK*β*, 1 : 1000; I*κ*B*α*, 1 : 1000; P65, 1 : 1000; MMP9, 1 : 1000; MMP13, 1 : 1000; PFKFB3, 1 : 1000; p-JNK, 1 : 1000; p-ERK, 1 : 1000; p-p38, 1 : 1000; p-IKK*α*/*β*, 1 : 500; p-I*κ*B*α*, 1 : 1000; p-P65, 1 : 500) incubated overnight at 4°C. Membranes were extensively washed in TBST for three times on the next day. The secondary antibody (anti-rabbit or anti-mouse IgG (H + L; 1 : 5000 dilution; Cell Signaling Technology, Danvers, MA, United States)) was subsequently incubated with the membranes for 1 hour at RT in the dark. Finally, the LI-COR Odyssey fluorescence imaging system was used to detect the reactivity (LI-COR Biosciences, Lincoln, NE, United States), and the ImageJ software was used to measure the grey values of each protein (National Institutes of Health, United States).

### 2.8. RNA Extraction and Real-Time Quantitative PCR (RT-qPCR) Analyses

To explore the effect of KAN on catabolic or anabolic-related genes, 3 × 10^5^ cells/well NPPs were cultured in DMEM on 6-well plates. 24 hours later, the NPPs were cultured with control, TNF-*α*, or TNF-*α* plus 2.5 *μ*M KAN for 24 hours. Total RNA was extracted using the Axygen RNA Miniprep Kit (Axygen, Union City, CA, USA) when the culture was completed. The Prime Script RT Master Mix kit was then used to reverse transcript the RNA to cDNA. Standard PCR (RT-qPCR) analyses were then performed as described previously [26]. Using the 2 − *ΔΔ*CT method, relative mRNA expression levels were calculated and normalized to the *β*-actin expression. All primer pairs (Table 1) were designed by NCBI BLAST.

### 2.9. Histology and Immunohistochemical Staining Analyses

After 4% paraformaldehyde fixing for 48 hours, IVD tissue samples were decalcification in the 10% EDTA for 21 days and then embedded into paraffin blocks. After being subjected to 5 *μ*m thickness histological sectioning, hematoxylin and eosin (H&E) staining and safranin O-fast green staining were then processed for histological assessment under standard laboratory protocols. All sections were captured using the high-quality microscope (Leica DM4000B). The histological score was evaluated based on the standard evaluation of 5 categories of degenerative changes [27]. For immunohistochemical evaluation, tissue sections were first dewaxed by graded xylene and standard alcohol gradients. After washing with PBS and water, 10% goat serum was used to block for 30 min at RT. Subsequently, the sections were incubated with the primary antibodies (anti–MMP9 and anti–MMP13 purchased from Servicebio, Wuhan, China) overnight at 4°C. The next day, the appropriate horseradish peroxidase labeled secondary antibody was incubated with the sections for 1 hour at RT and then developed with diaminobenzidine solution. Finally, all sections were captured under a high-quality microscope (Leica DM4000B).

### 2.10. Immunofluorescent Staining Analyses

Slices were processed of graded xylene deparaffinization, graded alcohol solution hydration, antigen retrieval, permeabilization, and blocking before immunofluorescent staining. Then, primary antibody incubation (anti-interleukin- (IL-) 1*β* and anti-collagen 2; dilution 1 : 100) (Cell Signaling Technology, Danvers, MA, United States; Abcam, Cambridge, United Kingdom), secondary antibody incubation, and nuclear staining were performed on these slides. The high-quality Leica DM4000 B epifluorescence microscope (Leica Microsystems GmbH) was used to observe and photograph the digital fluorescence images, and the ImageJ software was used to measure the IOD value.

### 2.11. Image Analysis and Disc Height Index Measurement

Cabinet X-ray imaging and irradiation systems (Faxitron Bioptics, LLC, Wheeling, IL, USA) were used to visualize the X-ray imaging of IVDs. Under identical acquisition parameters and imaging conditions, digital images were used to capture at 45 kVp. The disc height index of these IVDs was measured and normalized to sham group [28]. The calculated values were the average of three measurements per disc.

### 2.12. Animals and Surgical Procedures

The male Sprague–Dawley rats (Shanghai Lab, Animal Research Center Co., Ltd., China) used in this research were carefully housed (12 h day/night cycle, pathogen-free conditions, 26–28°C, and 50–65% humidity). When rats were housed to the age of eight weeks old, pentobarbital sodium (5 mg/100 g body weight) was used to anesthetize them by intraperitoneal injection before surgical procedures. The iodinated polyvinylpyrrolidone was first used for sterilization, and then, a ventral longitudinal skin incision was made over the tail. The revealed coccyx vertebrae (Co) 3–7 intervertebral discs were selected for experiments (the Co3/4 IVDs: sham controls; the Co4/5, Co5/6, and Co6/7 IVDs: a 20-gauge sterile needle-oriented perpendicular puncture to the skin at the center of the disc and rotating once to make sure insertion level through the AF, into the NP). Surgical exposure was repeated after one week recover under the sutured incision state, and Co4/5, Co5/6, and Co6/7 IVDs were separately injected with 50 *μ*L vehicle, low-dose KAN (2.5 *μ*M), and high-dose KAN (5 *μ*M). After incision suture and 4 weeks of recovery, all experiment rats were sacrificed. The tails were extracted and cleaned of soft tissues and then fixed in 4% PFA.

All animal experiments were authorized by the Institutional Animal Care and Ethics Committee of the Shanghai Ninth People's Hospital, Shanghai Jiaotong University School of Medicine. All animals were operated in accordance with the Guidelines for Animal Treatment of Shanghai Jiaotong University and the principles and procedures of the National Institutes of Health (NIH) Guide for the Care and Use of Laboratory Animals.

### 2.13. Statistical Analysis

All data were expressed in the form of mean ± standard deviation. The comparison between the experimental group and the control group was conducted by the 2-tailed, unpaired Student's *t*-test, or one-way ANOVA with Tukey's post hoc test. Moreover, significant differences in ordinal data between study groups were assessed by the Friedman test with Dunn's post hoc test. Statistical significance was calculated to be at no significant (ns); ^∗^*p* < 0.05; ^∗∗^*p* < 0.01; ^∗∗∗^*p* < 0.001; ^∗∗∗∗^*p* < 0.0001. All results were calculated and analyzed by Prism 8 (GraphPad Software Inc., San Diego, CA, USA).

## 3. Results

### 3.1. Effects of KAN on NPP Cytotoxicity and Proliferation

**Figure 1:**
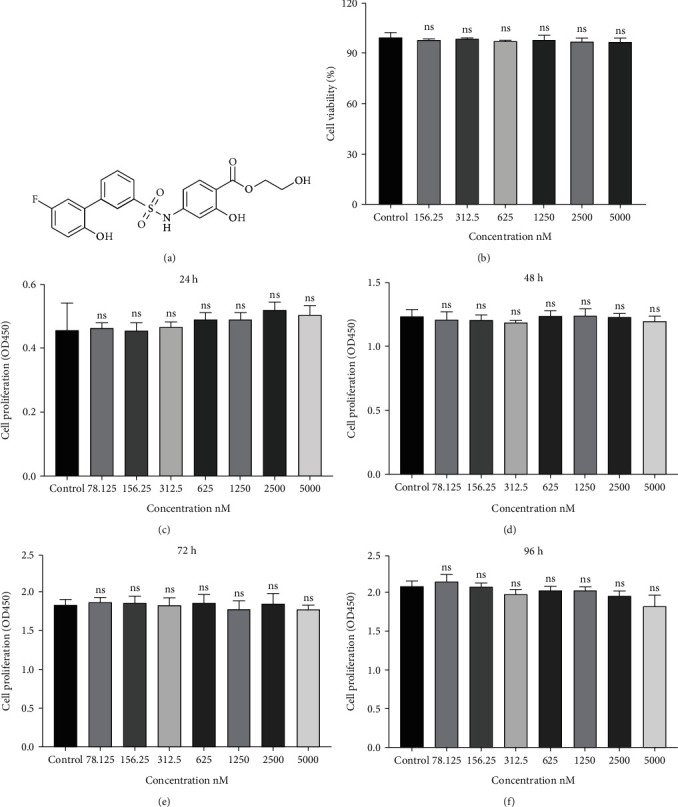
Effects of KAN0438757 (KAN) on the cytotoxicity and proliferation of nucleus pulposus primary (NPP) cells *in vitro*. (a) The chemical structure of KAN. (b) Cell viability of KAN-treated NPPs was tested by CCK-8 at 24 hours. (c–f) Cell proliferation effect of NPPs treated by negative control or different concentrations KAN was tested by CCK-8 at 24, 48, 72, and 96 hours. Data are expressed as mean ± SD, *n* = 4‐6. ns: no significance.

The KAN's chemical structure is presented in Figure 1(a). To investigate the proliferation and cytotoxicity effects of KAN on NPPs, the CCK-8 assay was performed under different cell numbers. 8,000/well NPPs were first seeded in 96-well plates and cultured for 24 h with various concentrations of KAN (control (0), 0.15625, 0.3125, 0.625, 1.25, 2.5, or 5 *μ*M) for cytotoxicity analysis, and no cytotoxicity was found in KAN under the concentration of 5 *μ*M (Figure 1(b)). For proliferation effects, NPPs were seeded at a density of 2,000/well in 96-well plates and cultured with various concentrations of KAN (control (0), 0.15625, 0.3125, 0.625, 1.25, 2.5, or 5 *μ*M) for 24, 48, 72, and 96 hours. The number of NPPs with any concentration of KAN has no significant variations compared with the control (0) group at any time point (Figures 1(c)–1(f)). Thus, the KAN has no effect on the proliferation of NPPs under the concentration of 5 *μ*M. According to the above experiments, 2.5 *μ*M of KAN was selected to be used in subsequent experiments.

### 3.2. KAN Alleviated TNF-*α*-Induced ECM Degradation of NPPs

**Figure 2:**
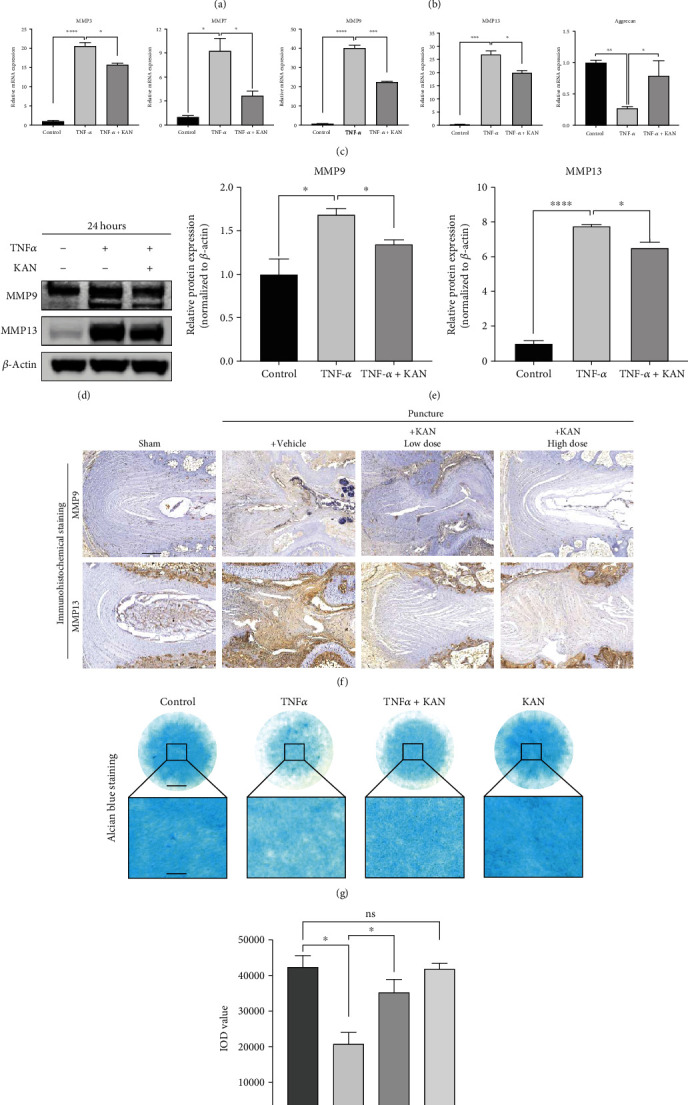
KAN0438757 (KAN) alleviated tumor necrosis factor-*α*- (TNF-*α*-) induced extracellular matrix (ECM) degradation of nucleus pulposus primary (NPP) cells both *in vitro* and *in vivo*. (a) PFKFB3 expression levels after negative control, TNF-*α*, TNF-*α* with 2.5 *μ*M KAN treatment for 24 hours in NPPs. (b) The gray levels of PFKFB3 were quantified and normalized to *β*-actin using ImageJ. *n* = 3. (c) Expression of the anabolism and catabolism gene matrix metalloproteins (MMPs) 3, 7, 9, and 13 and aggrecan after negative control, TNF-*α*, and TNF-*α* with 2.5 *μ*M KAN treatment for 24 hours in NPPs. *n* = 3. (d) MMP3 and MMP9 expression levels after negative control, TNF-*α*, and TNF-*α* with 2.5 *μ*M KAN treatment for 24 hours in NPPs. (e) The gray levels of MMP3 and MMP9 were quantified and normalized to *β*-actin using ImageJ. *n* = 3. (f) Immunohistochemical staining of MMP3 and MMP 9 expressions. Scale bar = 300 *μ*m. (g) Alcian blue staining of NP primary cells (P2 generation) on high-density culture after negative control, TNF-*α*, and TNF-*α* with 2.5 *μ*M KAN treatment for 7 days. Scale bar = 2 mm, 400 *μ*m. (h) The integrated optical density (IOD) value was calculated to evaluate the extracellular matrix (ECM) of NP primary cells after the indicated treatment. *n* = 4. Data are expressed as mean ± SD. ns: no significance; ^∗^*p* < 0.05; ^∗∗^*p* < 0.01; ^∗∗∗^*p* < 0.001; ^∗∗∗∗^*p* < 0.0001.

The inhibitor effect of KAN was firstly identified by significantly reduced protein expression of PFKFB3 in the treatment (TNF-*α* plus KAN) group compared with the upregulated expression in the TNF-*α* group (Figures 2(a) and 2(b)). To further explore KAN's role on ECM degradation, the anabolism and catabolism factors of ECM were detected by RT-qPCR *in vitro*. After that, the MMP3, MMP7, MMP9, and MMP13, which are catabolism-related genes, also partly recovered in mRNA levels after KAN treatment. Meanwhile, the mRNA expression of aggrecan, which is related to anabolism, also partly reversed in the treatment group (Figure 2(c)). Furthermore, the similar change in protein level detected by the WB experiment of MMP9 and MMP13 *in vitro* (Figures 2(d) and 2(e)) and the immunohistochemical staining of MMP9 and MMP13 *in vivo* (Figure 2(f)) further corroborated the results above. More intuitively, Alcian blue staining was used to reflect the variation of ECM after TNF-*α* induction and KAN treatment in NPPs. As shown in Figures 2(g) and 2(h), the indicated concentration KAN treatment reversed the ECM loss in the NPPs high-density culture induced by TNF-*α*, while no difference between the KAN alone group compared with the control group. In general, these results demonstrated that KAN alleviates TNF-*α*-induced ECM degradation of NPPs.

### 3.3. Effects of KAN on TNF-*α*-Induced NF-*κ*B and MAPK Signaling Pathway and Glycolysis Energy Metabolism Phenotype in NPPs

**Figure 3:**
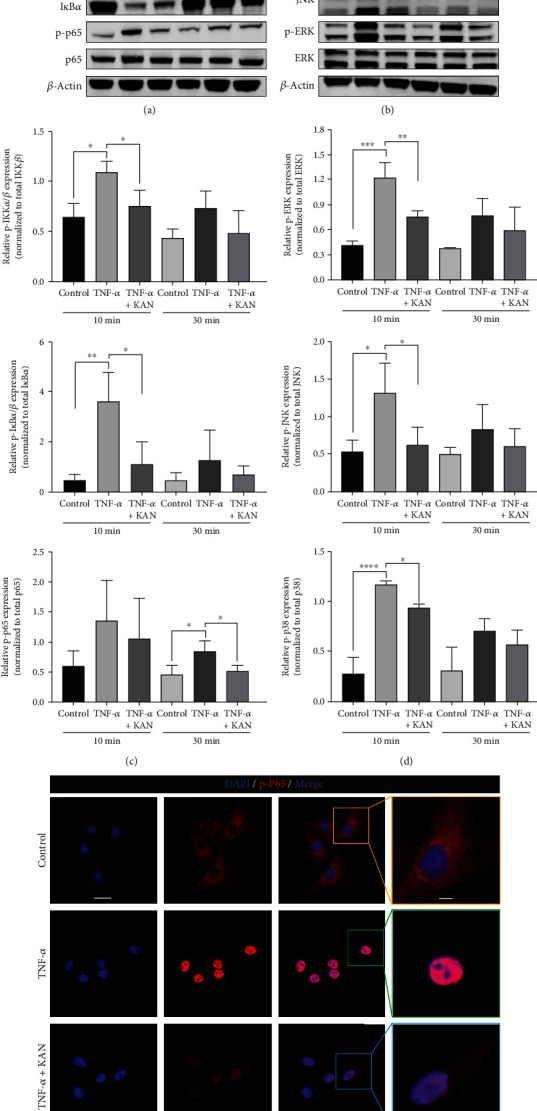
KAN0438757 (KAN) inhibited the NF-*κ*B signaling pathway and subsequent p-P65 nuclear translocation, as well as the MAPK signaling pathway. (a) The NF-*κ*B signaling pathway protein expression levels after negative control, TNF-*α*, and TNF-*α* with 2.5 *μ*M KAN treatment for 10 and 30 min in NPPs. (b) The MAPK signaling pathway protein expression levels after negative control, TNF-*α*, and TNF-*α* with 2.5 *μ*M KAN treatment for 10 and 30 min in NPPs. (c) The gray levels of p-IKK*α*/*β*, p-I*κ*B*α*, and p-P65 were quantified and normalized to their respective total protein using ImageJ. (d) The gray levels of p-ERK, p-JNK, and p-P38 were quantified and normalized to their respective total protein using ImageJ. (e) Nuclear translocation of p-P65 after negative control, TNF-*α*, and TNF-*α* with 2.5 *μ*M KAN treatment for 15 min, visualized by immunofluorescence. Scale bar = 20 *μ*m and 5 *μ*m. Data are expressed as mean ± SD, *n* = 3. ns: no significance; ^∗^*p* < 0.05; ^∗∗^*p* < 0.01; ^∗∗∗^*p* < 0.001; ^∗∗∗∗^*p* < 0.0001.

As previously reported, PFKFB3 inhibitor PFK15 could memorably repress inflammation (NF-*κ*B and MAPK signaling pathways) and block the glycolysis of osteoclasts simultaneously [23]. To investigate whether the underlying mechanisms of the KAN's effects on anabolism and catabolism on NPPs were similar to PFK15, NPPs were firstly pretreated with KAN for 2 hours and then treated with another 10 or 30 min of TNF-*α* stimulation. After that, we observed the classical NF-*κ*B signaling pathway transduction factor change, including the inhibitor of nuclear factor kappa B kinase (IKK), an inhibitor of nuclear factor kappa B (I*κ*B), and NF-*κ*B (p65). As we expected, the IKK, I*κ*B, and p65 phosphorylation were promoted with TNF-*α* induction while reversed after KAN treatment (Figures 3(a) and 3(c)). Moreover, immunofluorescence staining further showed that subsequent p-p65 nucleus translocation was also reversed after KAN treatment (Figure 3(e)). Meanwhile, another important signaling pathway mentioned, the MAPK signaling pathway, was also explored. Our western blot experiments demonstrated that the recovery effect of KAN was similarly observed in the phosphorylation of MAPK signaling pathway proteins, including JNK, p38, and extracellular signal-regulated kinases (ERKs) (Figures 3(b) and 3(d)). In a word, these results indicated that KAN could significantly reverse the TNF-*α*-induced NF-*κ*B and MAPK pathway activation in NPPs.

**Figure 4:**
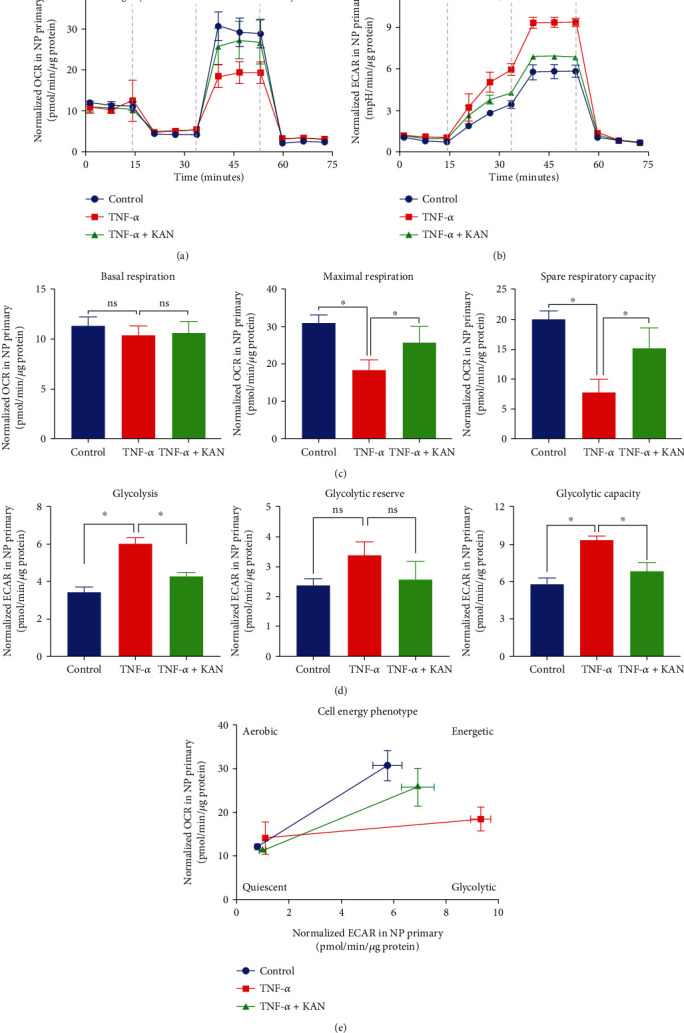
Metabolic reprogramming effects of KAN0438757 (KAN) on tumor necrosis factor-*α*- (TNF-*α*-) induced metabolism phenotype of nucleus pulposus primary (NPP) cells *in vitro*. (a) Normalized OCR of NPPs after negative control, TNF-*α*, and TNF-*α* with 2.5 *μ*M KAN treatment for 24 hours. (b) Normalized ECAR of NPPs after negative control, TNF-*α*, and TNF-*α* with 2.5 *μ*M KAN treatment for 24 hours. (c) Effects of KAN on basal respiration, maximal respiration, and spare respiratory capacity in OCR assay. (d) Effects of KAN on glycolysis, glycolytic capacity, and glycolytic reserve in ECAR assay. (e) Modified KAN reprogrammed the energy metabolism phenotype of TNF-*α*-inducted NPPs. Data are expressed as mean ± SD, *n* = 3. ns: no significance; ^∗^*p* < 0.05; ^∗∗^*p* < 0.01; ^∗∗∗^*p* < 0.001; ^∗∗∗∗^*p* < 0.0001.

Furthermore, the potential ability of KAN on NPP energy metabolism reprogramming was a novel direction that we totally interested in. Thus, the Seahorse extracellular flux analysis was performed to detect the OCR and ECAR of NPPs according to the instructions (Supplementary Figure 1). For the OCR assay, oligomycin, a mitochondrial ATP synthase inhibitor, was first used to block part of mitochondrial basal respiration and calculate ATP production. Next, the maximal and spare respiratory capacity was measured by FCCP via uncoupling mitochondrial respiration. Finally, the total mitochondria respiration was inhibited by rotenone and antimycin A and mitochondrial complexes I and III blocker, to display the nonmitochondrial oxygen consumption. For the ECAR analysis, enhanced ECAR value after glucose injection under the glucose-deficient environment indicated the glycolysis rate, while the further increase of ECAR value after injection of oligomycin represented the glycolytic reserve and the peak ECAR value meant glycolytic capacity. Similarly, the inhibitor of hexokinase, 2-deoxy-D-glucose (2-DG), totally blocked the glycolysis. Therefore, our results showed that TNF-*α* induction decreases the OCR of NPPs compared to normal conditions. However, the KAN treatment substantially rescued the OCR impairment caused by TNF-*α* induction. Specifically, KAN treatment reprogrammed the TNF-*α*-induced glycolysis energy metabolism phenotype of NPPs by promoting spare respiratory capacity and maximal respiration (Figures 4(a) and 4(c)). More obvious in the ECAR assay, TNF-*α* induction showed enhanced glycolysis rates and glycolytic capacity than normal conditions, which were also prominently suppressed by KAN treatment (Figures 4(b) and 4(d)). On the whole, all these data indicated that KAN treatment exactly reprogramed the TNF-*α*-induced glycolysis metabolic phenotype on NPPs (Figure 4(e)).

### 3.4. KAN Ameliorated IVDD Progression in the Puncture Injury Rat Model *In Vivo*

**Figure 5:**
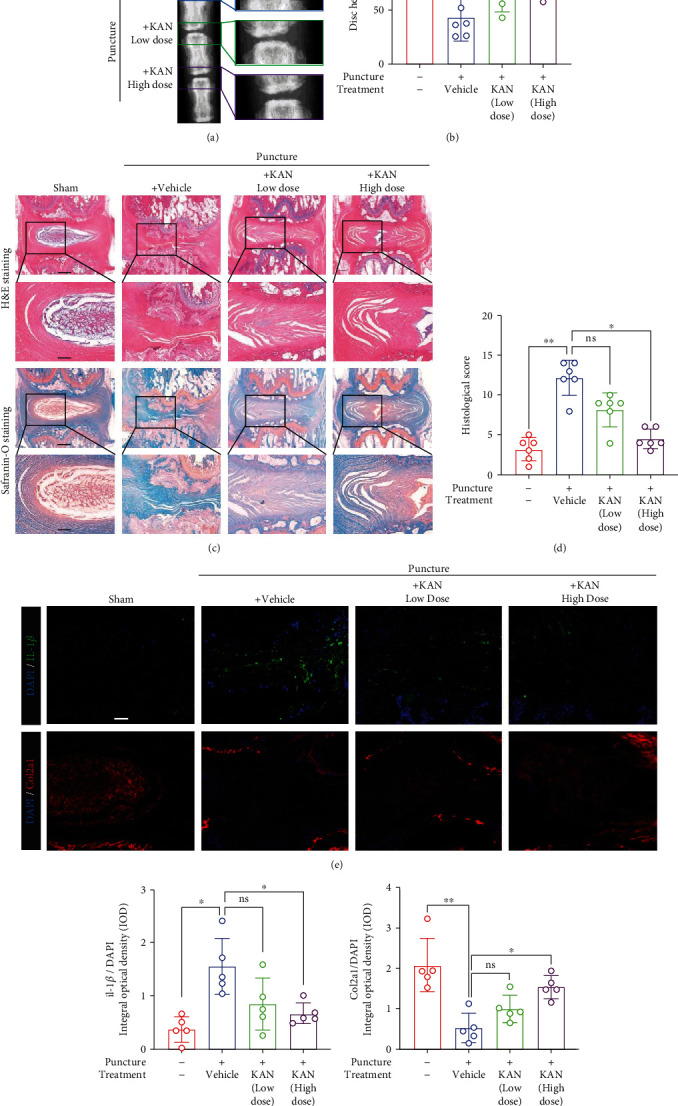
KAN0438757 (KAN) ameliorated the progression of intervertebral disc degeneration (IVDD) in a rat tail disc puncture model *in vivo*. (a) The rat coccyx vertebrae X-ray images at 4 weeks after treatment from each group. (b) Quantitative analysis of disc height index (DHI, %) of IVDs in mice at 4 weeks after treatment in each group. *n* = 6. (c) Hematoxylin and eosin staining and safranin O-fast green staining of intervertebral discs (IVDs) from each group at 4 weeks after treatment. Scale bar = 500 *μ*m and 200 *μ*m. (d) Quantitative analysis of histological scores of IVDs in mice at 4 weeks after treatment in each group. *n* = 6. (e) Interleukin- (IL-) 1*β* and Col2a1 immunofluorescence staining of IVDs from each group at 4 weeks after treatment. Scale bar = 100 *μ*m. (f, g) Quantitative analysis of IOD (IL-1*β* and Col2a1) of IVDs in mice at 4 weeks after treatment in each group. *n* = 5. Data are expressed as mean ± SD. ns: no significance; ^∗^*p* < 0.05; ^∗∗^*p* < 0.01; ^∗∗∗^*p* < 0.001; ^∗∗∗∗^*p* < 0.0001.

To explore the therapeutic potential of KAN on IVDD *in vivo*, we established IVDD model (Co 4–5, Co 5–6, and Co6–7) induced by puncture of rat tail intervertebral disc, and Co 3-4 was used as the sham operation group. Different concentrations of KAN were separately administered one week after injury to Co 5-6 and Co 6-7, and vehicle was injected to Co 4-5. As shown by 4 weeks' X-ray detection after treatment, the osteophyte of the vehicle group was obviously observed more than other groups. Moreover, vehicle group's disc height index (DHI score) was significantly lower than the sham group, while the KAN treatment memorably reversed the IVD height in a dose-dependent manner (Figures 5(a) and 5(b)). Therefore, KAN could reduce puncture-induced degeneration from the perspective of rat tail disc imaging detection. Similarly, the alleviated destruction of the IVD structure in the KAN treatment groups was also demonstrated by H&E staining and safranin O-fast green staining and the histological score (Figures 5(c) and 5(d)). Further immunofluorescence staining for IL-1*β* and Col2a1 was carried out to detect KAN's effect on inflammation and degradation of ECM *in vivo* (Figures 5(e)–5(g)). Coincidentally, the results corroborated that KAN substantially reverses the upregulation of IL-1*β* and the downregulation of Col2a1 in the tail disc tissue of rats. To sum up, these results demonstrated that KAN intervention is a promising potential therapeutic strategy for IVDD.

## 4. Discussion

IVD degeneration (IVDD) is one of the most common reasons for lumbar disc herniation, spinal stenosis, and subsequent lower back pain [29], which has imposed a huge socioeconomic burden. Nowadays, aging, abnormal biomechanical loading, acute trauma, decreased nutrient transport across the CEPs, smoking, genetic predisposition, and hyperglycemia have been corroborated as participating in the occurrence and development of IVDD [30]. Although NP cell senescence, subsequent ECM degradation, and triggered inflammation are considered to be the three main molecular mechanisms and therapeutic directions of IVDD [31], additional molecular mechanisms and underlying therapeutic targets of these causes are still unclear and need to be explored. In the last decade, acid environment change of IVD caused by aggravated glycolytic metabolism phenotype during IVDD, similar to hypoxia environment change, has been proved to participate and accelerate the whole processes of IVDD [12–16]. Thus, therapies targeting an enhanced glycolytic caused IVD acidic environment are a new direction worthy to be exploited. KAN, an emerging selective inhibitor of key rate-limiting metabolic kinase PFKFB3, has been used to treat colorectal cancer and elevate the efficacy of interstrand crosslink- (ICL-) inducing cancer therapies due to the function during DNA damage [32, 33]. However, research on the diseases principally influenced by its glycolysis regulation function is still blank. Moreover, considering the inhibition effect of PFK15, another PFKFB3 inhibitor, on glycolysis and inflammation (NF-*κ*B and MAPK pathways) during metabolic bone disease osteoporosis [23], our studies explored the role of KAN on energy metabolism reprogramming and inflammation in the process of IVDD.

Due to the properties of inflammatory cytokines, particularly interleukin 1*β* (IL-1*β*) and tumor necrosis factor-*α* (TNF-*α*), identified as a major risk factors for decreased NPP activity and extracellular matrix loss (including aggrecans) [34], TNF-*α*-induced NPP dysfunction and ECM degradation were selected to be studied in our present research. We demonstrated that nontoxic concentration KAN treatment could strongly reverse TNF-*α*-induced degradation of ECM. Specifically, after TNF-*α* induction, the upregulated MMP family (MMP3, MMP7, MMP9, and MMP13) mRNA expression related to catabolism [35] and the downregulation of Aggrecan related to anabolism [36] both partly recovered after KAN treatment. Our next western blot and immunohistochemical staining experiments of the MMP9 and MMP13 further authenticated the consistent change in protein level. Furthermore, NPP high-density culture which could observe the ECM variation more intuitively [37] showed that KAN treatment reversed the ECM loss after TNF-*α* stimulation. In general, we demonstrated the rescue effect of KAN on ECM degradation. The underlying mechanisms were also revealed. Although a lot of signaling pathways, such as NF-*κ*B, phosphatidylinositol-3-kinase (PI3K), MAPK, and Wnt signaling pathways, had been reported to mediate ECM metabolism or cell apoptosis after inflammation stimulation in the NPPs [38–41], we mainly focus on NF-*κ*B and MAPK signaling pathways that were reported on PFK15 research. According to the report about the decisive role of TNF-*α*-induced NF-*κ*B signaling pathway activation and subsequent p-p65 nuclear translocation in the onset and development of IVDD [42, 43], we performed western blot and immunofluorescence experiments to identify whether the above conclusion exists in our research and the results corroborated the inhibiting effect of KAN on NF-*κ*B signaling pathway activation and p-p65 nuclear translocation. Similarly, the repressing effect of KAN on the MAPK signaling that is important for ECM synthesis and degradation [44] was also demonstrated by the downregulated protein expressions of phosphorylated JNK, ERK, and p38. Taken together, our studies firstly authenticated the inhibiting effect of KAN on TNF-*α*-induced conventional NF-*κ*B and MAPK signaling pathways to alleviate the ECM degradation.

A more attractive role of KAN is the potential of reprogramming energy metabolism. Acid environment variation caused by NPPs aggravated glycolytic metabolism phenotype during IVDD had been demonstrated to enhance the lactate production and its efflux to ECM, which promoted the activation of NLRP3 inflammasome and the NPP extracellular matrix degeneration [45]. The terminal of persistent environment pH decline was the formation of a consistent vicious cycle that is environment acidification-inflammation initiate and ECM degradation-NPP senescence or death, which accelerates every process of IVDD. Therefore, energy metabolism reprogramming therapies are extremely valuable. In this research, we explored the explicit function of KAN treatment on energy metabolism reprogramming by the Seahorse extracellular flux analysis. The change of OCR and ECAR demonstrated the obvious glycolytic metabolism phenotype in TNF-*α*-induced NPPs. At the same time, KAN treatment partly reversed the reduction of spare respiratory capacity and maximal respiration and the increase of glycolysis rates and glycolytic capacity. All in all, our studies firstly corroborated KAN's energy metabolism reprogramming effect on TNF-*α*-induced glycolysis metabolic phenotype on NPPs.

Finally, we identified the therapeutic potential of KAN on the rat tail disc puncture-induced IVDD model *in vivo*. In our X-ray examination, the reduced DHI score of the vehicle group was memorably reversed in the high-dose KAN treatment group. Moreover, our H&E staining and safranin O-fast green staining showed more closer IVD structure of KAN treatment groups to the sham group, compared with the disordered structure of the vehicle group. Furthermore, immunofluorescence staining results manifested the inflammatory factor repression and ECM reservation effect of KAN *in vivo*. To sum up, these results demonstrated that KAN intervention is a promising potential therapeutic strategy for IVDD.

However, as the original research exploring the effect of KAN on NPPs and IVDD, we still have many limitations. Firstly, the intermediator between KAN and inflammation signaling pathways (NF-*κ*B and MAPK) remains to be revealed, and whether and how KAN influences other signaling pathways, such as PI3K, and Wnt signaling pathway is still unclear. Secondly, the ER stress [46], acid-sensing ion channel [45, 47], and transient receptor potential channel (TRPV4) [48, 49] are all influenced by pH and participated in the process of IVDD. Whether KAN regulated one or more of them to exert energy metabolism reprogramming and anti-inflammation effect during IVDD is worthy to be explored. Thirdly, Li et al. reported that nine cell types were identified in human normal nucleus pulposus (NNP) cells and human degenerative nucleus pulposus (DNP) by single-cell transcriptome sequencing [50]. According to the effect of PFKFB3 inhibition on ECs and OCs that had been proved in other research [20, 23], the inhibitory role of KAN on ECs and macrophages in NP could be partly forecasted. However, the research on the KAN effect on T cells, neutrophils, and different nucleus pulposus subsets is still blank and remains to be explored to better demonstrate whether it affects the process of human IVDD and its potential application value in humans. Moreover, the therapeutic effect of KAN on IVDD has only been explored and demonstrated in the rat tail intervertebral disc puncture model, which is not enough to achieve clinical application before the other IVDD models on rats, rabbits, and even monkeys have been authenticated.

## 5. Conclusion

**Figure 6:**
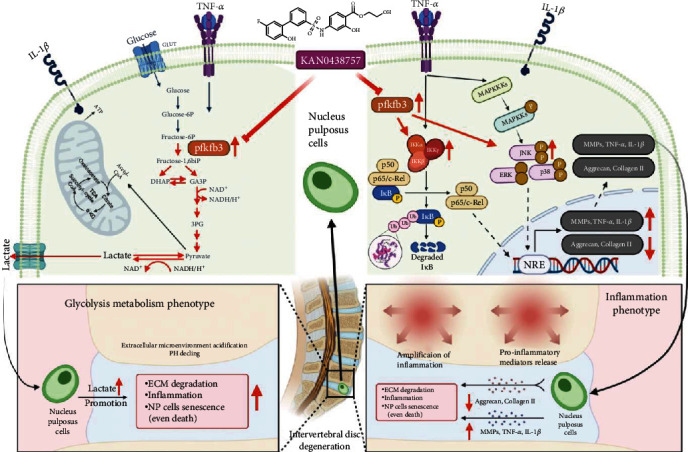
Schematic representation of the molecular mechanism of the article (created with http://BioRender.com).

Anyway, our research successfully demonstrated the potential therapeutic effect of KAN on IVDD and declaimed its mechanisms of both novel energy metabolism reprogramming and conventional anti-inflammation effect, which provided a fresh direction for researchers focusing on the influence of the IVD environment during the development of IVDD (Figures 6). Moreover, these results demonstrated that KAN intervention or PFKFB3 inhibition is a promising potential therapeutic strategy for IVDD in the future.

## Figures and Tables

**Table 1:** Primer pair sequences used in qPCR.

Mouse gene	Forward 5′ ➔ 3′	Reverse 5′**➔** 3′
*MMP3*	CCCTGCAACCGTGAAGAAGA	GACAGCATCCACCCTTGAGT
*MMP7*	CCCTGTTCTGCTTTGTGTGTCA	GGGGGAGAGTTTTCCAGTCA
*MMP9*	CCGACTTTTGTGGTCTTCCCC	ATGTCTCGCGGCAAGTCTTC
*MMP13*	AGAAGTGTGACCCAGCCCTA	GGTCACGGGATGGATGTTCA
*Aggrecan*	TGCAGACATTGACGAGTGCC	AGAGAGTGTCCGTCAGACCA
*β-Actin*	ACCCGCGAGTACAACCTTC	ATGCCGTGTTCAATGGGGTA

## Data Availability

The datasets used and/or analyzed during the current study are available from the corresponding author on reasonable request.

## Supplementary Materials

Supplementary Materials Supplementary Figure 1: OXPHOS characteristics and glycolysis parameter instructions and calculation methods. (A) OXPHOS characteristic instructions and calculation methods based on OCR assay. (B) Glycolysis parameter instructions and calculation methods based on ECAR assay.
