# Supplementary material for: Specific PFKFB3 Inhibitor Memorably Ameliorates Intervertebral Disc Degeneration via Inhibiting NF-κB and MAPK Signaling Pathway and Reprogramming of Energy Metabolism of Nucleus Pulposus Cells
Source: Oxid Med Cell Longev. 2022 Sep 21;2022:7548145. doi: 10.1155/2022/7548145 (PMC9519352; doi:10.1155/2022/7548145)
Supplement: Supplementary Materials — Supplementary Figure 1: OXPHOS characteristics and glycolysis parameter instructions and calculation methods. (A) OXPHOS characteristic instructions and calculation methods based on OCR assay. (B) Glycolysis parameter instructions and calculation methods based on ECAR assay. [file 7548145.f1.pdf]

A

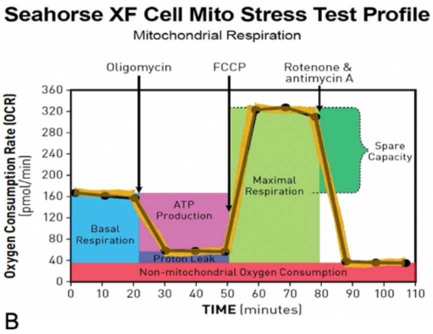

Parameter Value	Equation
Non-mitochondrial Oxygen Consumption	Minimum rate measurement after Rotenone/antimycin A injection
Basal Respiration	(Last rate measurement before first injection) – (Non-Mitochondrial Respiration Rate)
Maximal Respiration	(Maximum rate measurement after FCCP injection) – (Non-Mitochondrial Respiration)
H+ (Proton) Leak	(Minimum rate measurement after Oligomycin injection) – (Non-Mitochondrial Respiration
ATP Production	(Last rate measurement before Oligomycin injection) – (Minimum rate measurement after Oligomycin injection)
Spare Respiratory Capacity	(Maximal Respiration) – (Basal Respiration)
Spare Respiratory Capacity as a %	(Maximal Respiration) / (Basal Respiration) × 100
Acute Response	(Last rate measurement before oligomycin Injection) – (Last rate measurement before acute injection)
Coupling Efficiency	ATP Production Rate) / (Basal Respiration Rate) × 100

B

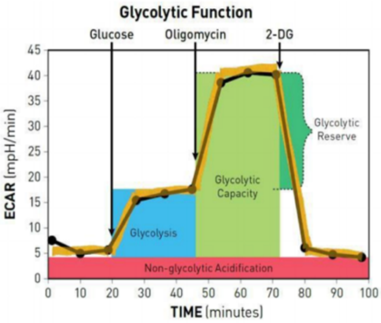

Parameter	Rate Measurement Equation Used by Report Generator
Glycolysis	(Maximum rate measurement before Oligomycin injection) – (Last rate measurement before Glucose injection)
Glycolytic Capacity	(Maximum rate measurement after Oligomycin injection) – (Last rate measurement before Glucose injection)
Glycolytic Reserve	(Glycolytic Capacity) – (Glycolysis)
Glycolytic Reserve as a %	(Glycolytic Capacity Rate) /(Glycolysis) × 100
Non-Glycolytic Acidification	Last rate measurement prior to glucose injection
Acute Response	(Last measurement rate before glucose injection – Last rate measurement before acute injection)
